# FoodCHOMP (Food Challenge—at HOme or in Medical Practice): a pilot multicentre randomised controlled trial evaluating home versus clinic-based food allergy challenges in low-risk adults–study protocol

**DOI:** 10.1136/bmjopen-2025-114483

**Published:** 2026-02-06

**Authors:** Jack Godsell, Sara Vogrin, Samantha Chan, Morgan Henri, Irvin Ng, Evelyn Andrews, Kymble Spriggs, Kirsten P Perrett, Jason Trubiano

**Affiliations:** 1 Department of Infectious Diseases and Immunology, Austin Health, Heidelberg, Victoria, Australia; 2 Department of Infectious Diseases, The Peter Doherty Institute for Infection and Immunity, Melbourne, Victoria, Australia; 3 Department of Clinical Immunology and Allergy, The Royal Melbourne Hospital, Melbourne, Victoria, Australia; 4 Centre for Antibiotic Allergy and Research, Department of Infectious Diseases and Immunology, Austin Health, Heidelberg, Victoria, Australia; 5 Infectious Diseases and Immunology, Austin Health, Heidelberg, Victoria, Australia; 6 Department of Allergy and Immunology, Monash Health, Clayton, Victoria, Australia; 7 National Allergy Centre of Excellence (NACE), Melbourne, Victoria, Australia; 8 Department of Medicine, The University of Melbourne Department of Medicine Royal Melbourne Hospital, Parkville, Victoria, Australia; 9 Department of Paediatrics, Murdoch Childrens Research Institute, Parkville, Victoria, Australia; 10 Department of Allergy and Immunology, The Royal Children’s Hospital Melbourne, Parkville, Victoria, Australia; 11 The University of Melbourne Department of Paediatrics, Parkville, Victoria, Australia; 12 Infectious Diseases, Austin Health, Heidelberg, Victoria, Australia; 13 The Peter Doherty Institute for Infection and Immunity, Melbourne, Victoria, Australia

**Keywords:** Allergy, IMMUNOLOGY, Clinical Trial

## Abstract

**Introduction:**

Approximately 6%–10% of adults carry food allergy labels. Many such labels are unverified and may be incorrect, contributing to delays in appropriate care, significant dietary restriction, anxiety and unnecessary use of emergency medications. Oral food challenges (OFCs) are the gold standard for confirming or excluding food allergy, but the current model of clinic-based challenges often has long wait times and logistical barriers. This study aims to investigate the feasibility and safety of home-based OFCs compared with standard in-clinic challenges in adults with negative skin-prick testing.

**Methods and analysis:**

**Food C**hallenge at **HO**me or in **M**edical **P**ractice is a pilot multicentre randomised controlled trial enrolling 120 adults with reported food allergy labels and negative skin prick testing to the implicated food. Participants will be randomised 1:1 to undergo an OFC either at home or in-clinic. The study is designed to generate feasibility and preliminary safety data for home-based OFC, measured by the proportion of participants experiencing immune-mediated adverse events (AEs), compared with clinic-based OFC. Feasibility metrics (screening to recruitment ratio, protocol completion), non-immune AEs, protocol adherence and quality of life assessed using the Food Allergy Quality of Life Questionnaire-12 at baseline prior to OFC and 6 months post OFC will also be collected. Statistical analysis will include descriptive statistics, with comparisons between arms using risk differences and relative risks with 95% CIs.

**Ethics and dissemination:**

The trial has received ethics approval from the Austin Health Human Research Ethics Committee (HREC/111750/Austin-2024). Findings will be disseminated through peer-reviewed publications and scientific meetings. Data will be presented in aggregated, de-identified form.

**Trial registration number:**

NCT06916819.

**STRENGTHS AND LIMITATIONS OF THIS STUDY**

- This is the first randomised controlled trial (RCT) evaluating the feasibility and safety of home-based food challenges compared with clinic oral food challenges (OFCs) in any population, with a particular focus on adults with negative skin prick testing.
- The study will provide feasibility data to inform a larger definitive trial of the efficacy of home food challenge as a ‘delabelling’ intervention.
- A limitation of the pilot feasibility and safety design is that the sample size will be underpowered for detecting rare severe adverse events.
- Results may not be generalisable beyond adult populations with negative skin-prick testing.
- The combination of registry and RCT data will provide a platform for future analysis of risk-predictors of positive OFC in adults.

## Introduction

 Patient-reported food allergies are a significant global health concern, with an estimated prevalence of 6%–10% among adults. The presence of a food allergy label can result in profound impacts on an individual’s quality of life, including anxiety, dietary restriction and the burden of carrying emergency medications such as epinephrine autoinjectors.^1^,^3^ Furthermore, unnecessary food avoidance may lead to nutritional deficiencies, social limitations and increased healthcare utilisation.^3 4^

Potential contributors to false food allergy labels include historical events in which a food was incorrectly identified as the trigger of symptoms and resolution of historical allergies with subsequent development of tolerance not yet recognised by the patient. A substantial proportion of patient-reported food allergies in adults are unverified and may be incorrect.^1^ Such labels are suspected to persist due to the absence of robust data to guide clinicians in challenging food allergy labels in adults, factors relating to delayed access to diagnostic food challenges or patient anxiety.

At present, there is no standard definition of a ‘low-risk’ adult patient-reported food allergy—one that is likely to be proven false on food challenge. The absence of sensitisation, as measured by negative skin prick testing (SPT) (<3 mm greater than the negative control) has been suggested to have sufficient sensitivity to ‘practically exclude’ food allergy; however, case literature exists of reactions to food challenge with negative skin testing prior.^5 6^

In this study, negative skin testing to the implicated allergen immediately prior to challenge is used to define a ‘low-risk’ patient reported food allergy appropriate for home challenge.

Despite advances in the performance of in vitro diagnostic methods such as basophil activation testing and component resolved diagnostics, a key limitation in current practice remains the challenge of timely and accurate confirmation of food allergy in vivo. The current gold standard for diagnosing food allergy is the double-blind, placebo-controlled food challenge (DBPCFC).^7^ However, DBPCFCs are resource-intensive and rarely feasible outside research settings.^8^ In clinical practice, open oral food challenges (OFCs) are widely employed to confirm or refute suspected allergies. Nevertheless, in-clinic food challenges pose significant logistical challenges, including the need for specialised facilities, trained staff and extended appointment durations.^8 9^

Even when food challenges are successfully performed in-clinic, up to 20%–40% of patients fail to reintroduce a challenge-tolerated food into their diet due to persistent anxiety or lack of confidence.^10^ This highlights a critical gap in translating negative challenge outcomes into meaningful changes in patient behaviour.

Home-based food challenges have been proposed as a potential strategy to improve access, reduce healthcare burdens and possibly enhance real-world reintroduction of foods.^711^,^13^ While guidelines and expert opinion have proposed criteria for safe home challenges, these recommendations are largely derived from paediatric populations, and there remains no prospective trial data assessing the feasibility or safety of home food challenges, particularly in adults.^11^ To date, no randomised controlled trials (RCTs) have directly compared home-based to clinic-based food challenges in any population.

The **Food C**hallenge at **HO**me or in **M**edical **P**ractice (FoodCHOMP) study aims to address this knowledge gap by evaluating the safety and feasibility of home-based food challenges in adults with low-risk food allergy labels. This pilot RCT seeks to generate critical data to inform the design of larger definitive trials and to explore whether home challenges may represent a viable and safe alternative to standard in-clinic testing for selected low-risk patients.

## Methods and analysis

### Study design

FoodCHOMP is a pilot multicentre, prospective, RCT designed to evaluate the safety and feasibility of home-based food challenges compared with standard in-clinic challenges. The trial will be conducted at two tertiary referral allergy centres in Melbourne, Australia: Austin Health and the Royal Melbourne Hospital.

All adult patients referred to the outpatient allergy services of these hospitals with a reported food allergy will undergo standard assessment including detailed clinical history and SPT as part of routine clinical practice. The requirement for endorsement of a referring clinician (eg, primary care, emergency physician) is to exclude spurious self-reported labels in the absence of a history of reaction. Patients with negative SPT to the implicated food allergen (weal size <3 mm greater than the negative control) will be assessed for trial eligibility. Blood collection for assessment of food allergen specific IgEs, where not previously available, will be collected at the time of recruitment; however, clinicians will remain blinded to these results at the time of randomisation.

Participants meeting inclusion criteria will be randomised in a 1:1 ratio to either a home-based OFC (intervention arm) or an in-clinic OFC (standard of care arm). The trial will enrol a total of 120 participants (60 per arm). The study is designed to generate feasibility and preliminary safety data to inform a larger definitive trial assessing non-inferiority of home-based food challenges.

The trial is registered with ClinicalTrials.gov (NCT06916819) and will adhere to the Consolidated Standards of Reporting Trials extension for pilot and feasibility trials.

### Eligibility criteria

Participants screened for the FoodCHOMP trial include all adults referred to outpatient allergy services with a reported food allergy label. Eligibility criteria were designed to maximise safety during food challenge procedures (Box 1). Inclusion criteria focus on individuals with negative SPT to the implicated food allergen at the time of screening and capacity to provide informed consent. Conversely, a range of exclusion criteria are applied to minimise risk, including the exclusion of specified medical conditions such as poorly controlled asthma, and use of medications that could interfere with challenge outcomes. These criteria are designed to identify a cohort considered low-risk for a severe allergic reaction and/or poor clinical outcome in the event of a severe allergic reaction (eg, uncontrolled asthma or advanced cardiac disease). Where participants have documentation of prior sensitisation to the selected food allergen (ie, previously positive skin tests or allergen specific IgE) skin testing was performed in duplicate. Patients with a reported food allergy label with a screen fail are eligible for the trial registry of the FoodCHOMP.

Box 1 **Inclusion and exclusion criteria**

*Inclusion criteria*

- Participants will be eligible for inclusion if they meet all of the following criteria:
- Aged 18 years or older.
- Referred to the outpatient allergy clinic with a reported food allergy label.
- Negative skin prick testing (weal <3 mm greater than the negative control) to the food implicated in their allergy label.
- Able and willing to provide informed consent.

*Exclusion criteria*

- Participants will be excluded if any of the following criteria are present:
- Pregnancy.
- Poorly controlled asthma, defined as an Asthma Control Questionnaire-5 score >1 at the time of enrolment.
- History of food reactions inconsistent with IgE-mediated processes (eg, exclusively gastrointestinal symptoms, food protein-induced enterocolitis syndrome).
- Clear history of food-dependent exercise-induced anaphylaxis.
- Current use of medications that may influence the outcome of the challenge, including:
- Antihistamine therapy.
- Systemic corticosteroids at doses exceeding stress-dose thresholds (ie, >50 mg hydrocortisone four times daily or equivalent).
- Omalizumab or alternate biologic therapy.
- Any other illness or condition that, in the investigator’s judgement, would substantially increase the risk associated with participation in the study.

### Recruitment and consent

Potential participants will be identified from referrals to the outpatient allergy services at participating hospitals. All patients referred with a reported food allergy label will undergo standard clinical assessment, including detailed allergy history and SPT.

Patients who meet eligibility criteria based on negative SPT will be approached by the research team to discuss participation in the trial. Verbal and written information about the study will be provided, and written informed consent will be obtained before participation.

Those who fail to meet eligibility criteria or consent will be included in a trial registry. A waiver of consent has been obtained for the collection of routine clinical data from standard allergy assessments for patients not enrolled in the randomisation arm to be included in the registry. This data includes demographics, information on all reported food allergy labels, general allergy history, comorbidities, current medications and previous allergy testing outcomes. Data will be collected in a de-identified format.

### Randomisation

Eligible participants will be randomised in a 1:1 ratio to either the intervention arm (home-based food challenge) or the control arm (standard in-clinic food challenge). Randomisation will be performed using a permuted block design, stratified by hospital site, participant gender and evidence of prior sensitisation to the challenge food, to ensure balanced allocation across study arms.

The randomisation sequence will be generated in Stata and delivered through REDCap (Research Electronic Data Capture), hosted on secure servers at Austin Health. Allocation concealment will be ensured until the point of randomisation. Investigators and participants will not be blinded to group assignment due to the nature of the intervention.

### Treatment arms

#### Home-based food challenge (Intervention arm)

Participants randomised to the intervention arm will undertake a home-based OFC following a structured protocol. An initial supervised dose of the implicated food will be administered in-clinic with a 60-minute observation period to assess for immediate reactions. If no reaction occurs, participants will proceed to continue the challenge at home.

**Figure 1:**
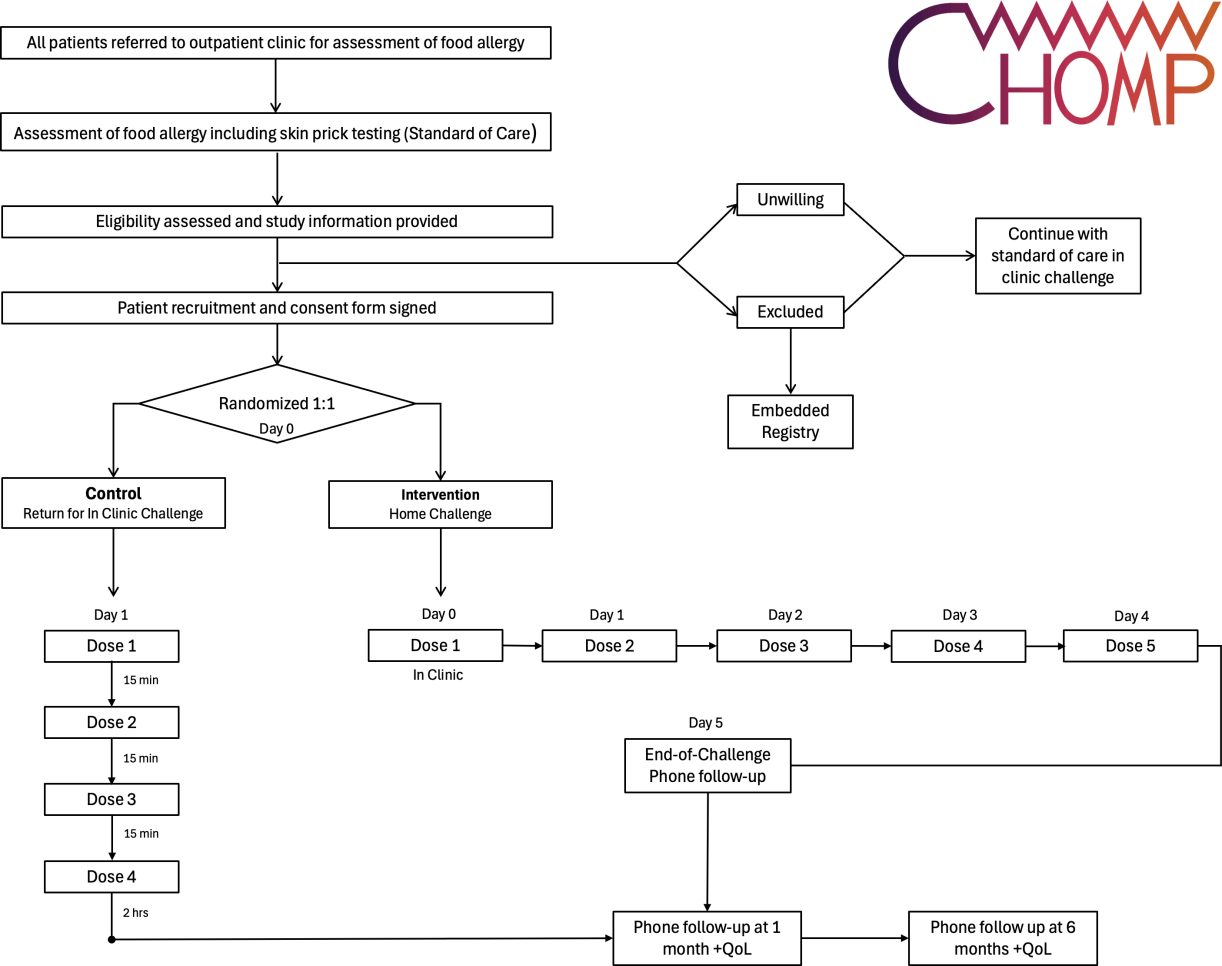
**Food C**hallenge at **HO**me or in **M**edical **P**ractice (FoodCHOMP) participant flow. Food Allergy Quality of Life (QoL) is assessed at 1 & 6 months.

The home challenge protocol involves graded daily dosing over 5 days, beginning with a small initial amount and increasing to a full serving by Day 5, provided no adverse reactions occur. The dosing schedule for the home challenge is detailed in figure 1 and doses are drawn from the Food Allergy Challenge Doses table (online supplemental material 1). Participants will receive written patient instructions outlining the protocol, symptom monitoring and emergency management (online supplemental material 2). Participants are advised to abstain from exercise for 2 hours following each dose. Participants are otherwise encouraged to continue their typical occupation during the challenge period with restriction.

Participants will be advised to cease the challenge immediately if symptoms occur and to follow their prescribed standardised Australian Society for Clinical Immunology & Allergy (ASCIA) Action Plan for Allergic Reactions or Anaphylaxis. Contact details for the research team will be provided for reporting adverse events (AEs).

Participants will be eligible to have one food allergy label assessed through this trial. Where an individual has multiple eligible food allergy labels, the selection of the food allergen to be investigated within this trial will be at the discretion of the participant. Further food allergy labels that remain at the end of the trial will be assessed as per standard of care.

#### In-clinic food challenge (Control arm)

Participants randomised to the control arm will undergo a standard in-clinic OFC, following existing protocols at participating sites. Challenges are performed using a four-step protocol adapted from the PRACTALL^5^ guidelines, with incremental doses administered at regular intervals over several hours under direct medical supervision.^5^ The dosing schedule for the in-clinic challenge is presented in figure 1 and doses are drawn from the Food Allergy Challenge Doses table (online supplemental material 1). Participants will be observed for at least 2 hours after the final dose to monitor for delayed reactions.

In both study arms, participants will be provided with an ASCIA Action Plan if clinically indicated and, where appropriate, prescribed an epinephrine device for use in the event of severe reactions.

### Outcomes

The primary outcome is the safety of home-based OFC, measured by the proportion of participants who experience an immune-mediated AE during the food challenge. Immune-mediated AEs are defined as symptoms indicative of an IgE-mediated hypersensitivity reaction to the challenge food, as defined by the PRACTALL guidelines.^9^ AEs will be reviewed to determine the severity and causality (immune-mediated vs non-immune-mediated) by an independent panel consisting of two allergy specialists from non-participating sites. In the event of discordant assessment between the two allergists, the decision will be passed onto a third clinician.

In addition to the primary safety outcome, the study will assess a range of secondary outcomes evaluating recruitment feasibility, protocol adherence and the occurrence of non-immune mediated AEs (Box 2). These measures will help determine whether home-based challenges are practical and acceptable in routine clinical practice. Furthermore, exploratory outcomes will investigate patient-reported quality of life, rates of sustained food reintroduction and potential health economic impacts, providing insights into the broader clinical and societal implications of adopting home food challenges.

Box 2 **Secondary and exploratory outcomes**

*Secondary outcomes*

- Secondary outcomes include:
- Proportion of participants who experience non-immune-mediated adverse events during the food challenge, including:
- Taste aversion
- Anxiety preventing completion of the challenge.
- Feasibility outcome measures:
- Eligibility to screened ratio (*proportion of screened patients who meet eligibility criteria*).
- Recruitment to eligibility ratio (*proportion of eligible patients willing to participate*).
- Intervention to recruitment ratio (*proportion of recruited patients who complete oral food challenge*).
- Identification of barriers to protocol completion in the home challenge arm, assessed through participant interviews.
- Protocol compliance in both study arms.
- Utility outcome:
- Proportion of participants identified as tolerant to the implicated food after challenge.

*Exploratory outcomes*

- Exploratory outcomes include:
- Change in food allergy-related quality of life, assessed using the Food Allergy Quality of Life Questionnaire-12 at baseline, 1 month and 6 months postchallenge (online supplemental material 3).
- Degree of active consumption of the implicated food following a negative challenge result 1 month and 6 months postchallenge.
- Time from referral to completion of food challenge.
- Health economic impact of home-based compared with in-clinic food challenges.

Participants in both arms will undergo baseline, 1-month and 6-month food allergy quality of life assessment using the FAQLQ-12 and assessment of degree of consumption of the challenged food (online supplemental material 3).^14^

### Sample size and statistical analysis

A total of 120 participants (60 per arm) will be recruited for this pilot trial. This sample size will provide estimates of safety and recruitment feasibility outcomes with a precision of <20% (total width of CIs). The chosen sample size is also considered sufficient to generate reliable estimates of safety and potential efficacy for planning a future definitive trial, as precision gains diminish beyond approximately 60 participants per group for binary outcomes.^15^

All analyses will follow the intention-to-treat principle, with supplementary per-protocol analyses conducted for sensitivity. Continuous variables will be summarised as medians with IQRs, while categorical variables will be presented as frequencies and percentages.

Binary outcomes will be presented as counts and proportions with exact 95% CIs. Comparative analyses between study arms will report absolute risk differences and relative risks, each with 95% CIs, using generalised linear models with a binomial family and identity or log link functions as appropriate.

No interim analyses are planned for this pilot study. Missing data are expected to be minimal and, where necessary, will be described in reporting.

### Data management

Participant clinical data and demographic information will be initially recorded in the electronic medical record systems at participating sites. Relevant study data will then be transferred into REDCap databases hosted on secure, password-protected servers at Austin Health. Data from each site will be stored locally, with a master database maintained centrally at Austin Health for analysis.

All data related to this study will be retained for 15 years after study completion, in accordance with institutional policies, after which all electronic records will be securely destroyed.

Only aggregated, non-identifiable data will be presented in publications and reports.

### Adverse events

AEs in this study are categorised as immune-mediated, non-immune-mediated or serious AEs.

Immune-mediated AEs are defined as symptoms consistent with an IgE-mediated allergic reaction occurring during or within 48 hours of the OFC. These may include, but are not limited to, cutaneous symptoms such as urticaria or angioedema, gastrointestinal symptoms such as vomiting or diarrhoea, or respiratory symptoms consistent with anaphylaxis. Such events will be classified according to criteria outlined in the PRACTALL guidelines and will be adjudicated by two independent reviewers, with a third reviewer as a tie-breaker where necessary.^9^

Non-immune-mediated AEs refer to symptoms that are not indicative of IgE-mediated allergy and do not meet criteria for a serious AE. Examples include taste aversion or anxiety leading to discontinuation of the challenge, nausea or gastrointestinal discomfort unrelated to an allergic mechanism. These will also be assessed within 48 hours of the food challenge.

A serious adverse event is defined as any untoward medical occurrence that, in the opinion of the independent adjudicators, is causally related to the food challenge and results in one or more of the following outcomes: death; life-threatening reaction; inpatient hospitalisation; persistent or significant disability or incapacity or an event requiring intervention to prevent permanent impairment or damage.

### Withdrawals and stopping criteria

Participants may withdraw from the study at any time without providing a reason. Data collected prior to withdrawal may be included in the final analyses unless the participant specifically requests that their data be destroyed and it has not yet been incorporated into the analysis. No participants who withdraw after randomisation will be replaced.

### Patient and public involvement

Patients and the public were actively involved in the design and development of this trial. Allergy and Anaphylaxis Australia, the largest consumer advocacy organisation for people with allergies in Australia, provided feedback on the trial design, participant-facing materials and study documentation.

Patients will not be directly involved in recruitment or conduct of the trial but will be invited to contribute to the dissemination of study findings through collaboration with consumer advocacy groups.

### Ethics and dissemination

This study will be conducted in accordance with the Declaration of Helsinki, the National Health and Medical Research Council National Statement on Ethical Conduct in Human Research (2007, updated 2018), and the Australian Code for the Responsible Conduct of Research (2018).

Ethics approval has been obtained from the Austin Health Human Research Ethics Committee (HREC/111750/Austin-2024). All participants will provide written informed consent before enrolment.

The results of this study will be disseminated through peer-reviewed publications, conference presentations and collaboration with consumer advocacy groups. Only aggregated, de-identified data will be reported, ensuring participant confidentiality.

## Supplementary material

10.1136/bmjopen-2025-114483 online supplemental file 1

10.1136/bmjopen-2025-114483 online supplemental file 2

10.1136/bmjopen-2025-114483 online supplemental file 3
