# Supplementary material for: FoodCHOMP (Food Challenge—at HOme or in Medical Practice): a pilot multicentre randomised controlled trial evaluating home versus clinic-based food allergy challenges in low-risk adults–study protocol
Source: BMJ Open. 2026 Feb 6;16(2):e114483. doi: 10.1136/bmjopen-2025-114483 (PMC12887465; doi:10.1136/bmjopen-2025-114483)
Supplement: online supplemental file 1 [file bmjopen-16-2-s001.docx]

SUPPLEMENTARY MATERIAL 1 – Food Allergy Challenge Doses

| **Home Challenge** | **Day 0 – Step 1** | **Day 1 – Step 2** | **Day 2 – Step 3** | **Day 3 – Step 4** | **Day 4 – Step 5** |
| --- | --- | --- | --- | --- | --- |
| **In-Clinic Challenge** | **Step 1** | **Step 2** | **Step 3** | **Step 4** |  |
| **Nuts** |  |  |  |  |  |
| Peanuts | 2 nuts | 5 nuts | 7 nuts | 14 nuts | 28 nuts |
| Almond | 1 nut | 4 nuts | 6 nuts | 12 nuts | 23 nuts |
| Cashew | 1 nut | 3 nuts | 5 nuts | 9 nuts | 18 nuts |
| Hazelnut | 1 nuts | 4 nuts | 5 nuts | 11 nuts | 21 nuts |
| Pistachio | 3 nuts | 7 nuts | 11 nuts | 23 nuts | 44 whole nuts |
| Walnut | 1 half | 3 halves | 4 halves | 7 halves | 14 halves |
| Pecans | 1 half | 3 halves | 7 halves | 11 halves | 22 halves |
| Brazil nut | ½ nut | 1 nut | 1 ½ nuts | 3 nuts | 6 nuts |
| Macadamia | 1 ½ nuts | 2 ½ nuts | 4 nuts | 8 nuts | 16 nuts |
| Pine nut | 2g | 4g | 9g | 15g | 30g |
| **Proteins** |  |  |  |  |  |
| Fish/Shellfish/Meat | 10g | 15g | 25g | 50g | 100g |
| **Other** |  |  |  |  |  |
| Wheat (Cooked Risoni) | 10g | 17g | 25g | 50g | 100g |
| Scrambled Egg | 1/12 egg | 1/6 egg | ¼ egg | ½ egg | 1 egg |
| Milk | 1 tbsp | 2 tbsp | 60ml | 125ml | 250ml |
| Soy Milk | 1 tbsp | 2 tbsp | 60ml | 125ml | 250ml |
| Sesame (tahini) | 1/2 tsp | 1 tsp | 2 tsp | 4 tsp | 2 Tbsp |
| Fruit | 1 tbsp | 2 tbsp | ¼ cup | ½ cup | 1 cup |
| Vegetable | 1 tbsp | 2 tbsp | ¼ cup | ½ cup | 1 cup |
