# Supplementary material for: FoodCHOMP (Food Challenge—at HOme or in Medical Practice): a pilot multicentre randomised controlled trial evaluating home versus clinic-based food allergy challenges in low-risk adults–study protocol
Source: BMJ Open. 2026 Feb 6;16(2):e114483. doi: 10.1136/bmjopen-2025-114483 (PMC12887465; doi:10.1136/bmjopen-2025-114483)
Supplement: online supplemental file 2 [file bmjopen-16-2-s002.docx]

SUPPLEMENTARY MATERIAL 2 – Home Challenge Protocol

CHOMP - Home food challenge protocol

For patients who are currently avoiding a food or food-group because of suspected allergy, reintroduction of the food into the diet can be stressful. The reintroduction of food at home is sometimes called a ‘home challenge’. The intent of a ‘home challenge’ is to build confidence reintroducing a food into a patient’s diet when the suspicion of allergy is low.

When introducing a new food in the home, make sure you’re prepared for any unexpected allergic reaction by doing the following:

- Start your home introduction when you’re feeling well
- Eat the suspected food during daylight hours – not at night just before bed – so that you can observe any unexpected reaction.
- Give one dose of food each day, starting with a small amount of the food then trying a little more each day as described below
- Make sure you have access to your emergency medication and ASCIA Action Plan for Anaphylaxis or Allergic Reactions as appropriate – either antihistamines or an adrenaline auto-injector if you have been prescribed one.
- Watch for any signs of allergic reaction over one to two hours after eating the food. These include but are not limited to;
  - Itchy skin rash (such as hives or eczema flare) or swelling
  - Vomiting or diarrhoea
  - Anaphylaxis (persistent coughing, wheezing or difficult breathing, dizziness or collapse).

| **Name:** | **Challenge Food :** |  |  |
| --- | --- | --- | --- |
|  |  |  |  |
| **DOB:** | **Day 1** |  |  |
|  | **Day 2** |  |  |
| **URN:** | **Day 3** |  |  |
|  | **Day 4** |  |  |
|  | **Day 5** |  |  |

Once you have eaten a “full serve” of the food on Day 5 without reaction, you are considered to have passed your home challenge, and should continue to include that food item in your diet at least 2-3 times per week.

ADVERSE REACTIONS

If there is concern at any point that a reaction is occurring:

- Stop eating the food
- Follow your ASCIA ‘Allergic Reaction Action Plan’ or ‘Anaphylaxis Action Plan’
  - Anti-histamines (e.g. Zyrtec, Telfast) may be appropriate for mild reactions (e.g. hives, itch)
  - **If you have difficult/noisy breathing, tongue swelling, throat tightness, difficulty talking, persistent wheeze or feel light-headed, administer your Epipen**® **or Anapen**® **and call 000**
- When safe to do so, document:
  - The most recent amount of the new food
  - The amount of time between eating the food and reaction
  - The length of time the reaction lasted
  - Features of the reaction (e.g. hives, swelling, nausea, diarrhoea)
  - Whether any medications were taken
  - Take photos of the reaction if possible (such as rashes or swelling)
- Make contact with the research team on XXXX XXX XXX to inform us of the reaction – we will then make contact to obtain further information
