# Supplementary material for: FoodCHOMP (Food Challenge—at HOme or in Medical Practice): a pilot multicentre randomised controlled trial evaluating home versus clinic-based food allergy challenges in low-risk adults–study protocol
Source: BMJ Open. 2026 Feb 6;16(2):e114483. doi: 10.1136/bmjopen-2025-114483 (PMC12887465; doi:10.1136/bmjopen-2025-114483)
Supplement: online supplemental file 3 [file bmjopen-16-2-s003.docx]

SUPPLEMENTARY MATERIAL 3 – Short Food Allergy Quality of Life Questionnaire (FAQLQ-12)

| 0 | 1 | 2 | 3 | 4 | 5 | 6 |
| --- | --- | --- | --- | --- | --- | --- |
| Not | Barely | Slightly | Moderately | Quite | Very | Extremely |

**How *troublesome* do you find it, because of your food allergy, that you ...**

1. Must always be alert as to what you are eating?
2. Are less able to taste or try various products when eating out?
3. Must personally check whether you can eat something when eating out?
4. That the ingredients of a product change?

**How *troublesome* is it, because of your food allergy ...**

1. That labels are incomplete?
2. That people underestimate your problems caused by food allergy?
3. That it is unclear to which foods you are allergic?

**How *worried* are you because of your food allergy ...**

1. About your health?
2. That the allergic reactions to foods will become increasingly severe?

**How *frightened* are you because of your food allergy ...**

1. Of an allergic reaction?
2. Of accidentally eating the wrong food?
3. Of an allergic reaction when eating out despite the fact that your dietary restrictions have been discussed beforehand?

Reproduced from Coelho, JACI-IP, 2023

(1)

References:

1. Coelho GL de H, Lloyd M, Tang MLK, DunnGalvin A. The Short Food Allergy Quality of Life Questionnaire (FAQLQ-12) for Adults. J Allergy Clin Immunol: Pr. 2023;11(5):1522-1527.e5.
