# Supplementary material for: FoodCHOMP (Food Challenge—at HOme or in Medical Practice): a pilot multicentre randomised controlled trial evaluating home versus clinic-based food allergy challenges in low-risk adults–study protocol
Source: BMJ Open. 2026 Feb 6;16(2):e114483. doi: 10.1136/bmjopen-2025-114483 (PMC12887465; doi:10.1136/bmjopen-2025-114483)
Supplement: Supplementary file 4 [file bmjopen-2025-114483.reviewer_comments.pdf]

PEER REVIEW HISTORY

BMJ Open publishes all reviews undertaken for accepted manuscripts. Reviewers are asked to complete a checklist review form and are provided with free text boxes to elaborate on their assessment. These free text comments are reproduced below.

ARTICLE DETAILS

Title (Provisional)

FoodCHOMP (Food Challenge -at HOme or in Medical Practice) -a pilot multicentre randomised controlled trial evaluating home versus clinic-based food allergy challenges in low-risk adults: Study Protocol

Authors

Godsell, Jack; Vogrin, Sara; Chan, Samantha; Henri, Morgan; Ng, Irvin; Andrews, Evelyn; Spriggs, Kymble; Perrett, Kirsten P; Trubiano, Jason

---

VERSION 1 - REVIEW

**Reviewer**	**1**
**Name**	**O'Shea, Kelly M**
**Affiliation**	**University of Michigan**
**Date**	**10-Dec-2025**
**COI**	**Grant support received from DBV Technologies, Novartis Pharmaceuticals, ALK, Abbott Nutrition, ARS Pharmaceuticals.**

Thank you for the opportunity to review this manuscript. This study is well-designed and represents an important first step toward building an evidence base for adult home OFCs. This is a much-needed contribution given the logistical constraints of in-office OFCs.

MAJOR COMMENTS:

- It is not clear if patients who only have symptoms consistent with pollen food allergy syndrome will be excluded or included. I would recommend exclusion of this population as it will confound results. This population is more likely to have a negative SPT but will still experience symptoms upon challenge, leading to higher rates of immune-mediated events.

- In the patient instructions for home OFCs, for safety purposes, I recommend specifying that exercise is not recommended for 2 hours after the dose until the full challenge has been completed. After a successful challenge, then exercise after eating the food is not restricted.

MINOR COMMENTS:

- PAGE 5 LINE 41: 'lale' should be 'label'

- How will the study team verify the pregnancy exclusion? Are there plans to perform UPTs on all participants?

- PAGE 5 LINE 10-11: Previous IgE testing may not be accurate or representative of current IgE status for many patients. Consider adding a specified timeframe for use of previous IgE results (ie within the past 12 months)

**Reviewer**	**2**
**Name**	**Berghi, Ovidiu**
**Affiliation**	**Saint Mary Clinics and Laboratories**
**Date**	**20-Dec-2025**
**COI**	**None**

Dear Authors,

The study presents a good intention. Many people are diagnosed wrong by other specialist or self-diagnose with food allergy. Your intention to clarify the diagnostic in the way proposed in the material shall help people to rule out food allergy. I have two questions - the patients shall be recruited only from Melbourne or from a larger area? If a severe reaction appears in how many minutes can arrive an emergency team? The second questions Home-Based Food Challenge (Intervention Arm) means that the patients will stay at home for 4 days. How do you convince them to leave their academic or professional life? Because this days means they have to tahe a pause form the job with possible consequencies

---

**Reviewer**	**3**
**Name**	**Wojsyk-Banaszak, Irena**
**Affiliation**	**Poznan University of Medical Sciences**
**Date**	**22-Dec-2025**
**COI**	**None**

I have read with great interest a study protocol for: FoodCHOMP (Food Challenge -at HOme or in Medical Practice) -a pilot multicentre randomised controlled trial evaluating home versus clinic-based food allergy challenges in low-risk adults” authored by Godsell Jack and colleagues. The study has been designed to answer a very important topic of delabelling individuals with diagnosed/suspected non-IgE mediated food allergy, who avoid allegedly culprit food in their diet. Therefore the effort, that Authors plan to undertake, is especially worth appraisal.

The main question addressed by the research is the evaluation of feasibility of oral food challenge (OFC) outside the hospital setting.

The topic is relevant to the field since OFCs, despite being the gold standard in the diagnosis of food allergy are time- and resources consuming, which might result in undue delays in implementing the proper management..

The protocol is properly composed, and the aims of the study are clear. . The references are up to date and appropriate.

I have some minor concerns that should be addressed by the Authors

1. Will the patients have Epinephrine prescribed? – This should be clearly stated. If not all the patients will have the Epinephrine prescribed please state the reasons for prescribing or not.

2. I would suggest a phone call 48 hours after first dose and last dose ingestion to cover all possible AEs

3. How would the patients avoiding multiple foods be handled? Who and how would chose the product for OFC in these cases?

VERSION 1 - AUTHOR RESPONSE

Reviewer 1

- MAJOR COMMENTS #1:
    - *It is not clear if patients who only have symptoms consistent with pollen food allergy syndrome will be excluded or included. I would recommend exclusion of this population as it will confound results. This population is more likely to have a negative SPT but will still experience symptoms upon challenge, leading to higher rates of immune-mediated events.*

Response:

- We appreciate the reviewer highlighting this point and agree that this population has a higher likelihood of reacting on oral food challenge, even when extract skin prick testing is negative.
- Acknowledging the very valid reasons for recommending exclusion of this cohort, the intent of the protocol is that these patients are included, for the following reasons:
    - Prior to challenge, this patient population will frequently be difficult to distinguish from true IgE-mediated allergy to the fruit, vegetable or nut in question. Prick-to-prick testing will be performed for all participants prior to challenge in to address the risk of false negatives to extract skin testing

        - This population represents significant real-world cohort in which home food challenges are likely to be implemented.
    - We agree with the reviewer that this may result in a higher rate of immune mediated events. To address the risk of confounding, participants who, on challenge, are found to have isolated oral symptoms consistent with PFAS will be specified across both arms.
    - Furthermore, as this is a pilot safety and feasibility study, the manner in which these reactions occur and are managed will inform the subsequent planned follow-on study.
- MAJOR COMMENTS #2
    - *In the patient instructions for home OFCs, for safety purposes, I recommend specifying that exercise is not recommended for 2 hours after the dose until the full challenge has been completed. After a successful challenge, then exercise after eating the food is not restricted.*

Response:

- We appreciate this feedback and will now included this in both written in the home challenge instructions and verbally in the counselling that patient receive. This will be included in the protocol manual and published as an appendix in the trial manuscript.

- MINOR COMMENTS:
    - PAGE 5 LINE 41: 'lale' should be 'label'
        - Thank you for highlighting this; it has now been corrected
    - *How will the study team verify the pregnancy exclusion? Are there plans to perform UPTs on all participants?*
        - At present, there are no plans to verify pregnancy status as an exclusion. A pragmatic approach has been taken, similar to standard-of-care in-clinic challenges, where patient disclosure of current pregnancy is satisfactory to preclude the food challenge. The protocol has been amended to clarify this point.
    - *PAGE 5 LINE 10-11: Previous IgE testing may not be accurate or representative of current IgE status for many patients. Consider adding a specified timeframe for use of previous IgE results (ie within the past 12 months)*
        - We agree with the reviewer that previous IgE testing, and particularly distant results, are likely to be inaccurate.
        - Participants will be stratified based on a history of ever having a positive marker of sensitization (either SPT or positive spIgE), to ensure representation of this group across both arms.

- Our hypothesis is that serum specific IgE testing is a poor predictor of outcome of food challenge in an individual with negative skin prick testing; this will be explored at further in the second phase of this trial, following on from the pilot safety and feasibility work.

Reviewer 2

1. *Patients shall be recruited only from Melbourne or from a larger area? If a severe reaction appears in how many minutes can arrive an emergency team?*
   - Participants are recruited from within the catchments of both Royal Melbourne and Austin Health, which extend out into rural and regional Victoria. The Statewide Ambulance Victoria response times for a critical medical condition like anaphylaxis average 15 minutes across the state. For non-urgent concerns, a direct mobile phone contact is provided to all participants.
2. *The second questions Home-Based Food Challenge (Intervention Arm) means that the patients will stay at home for 4 days. How do you convince them to leave their academic or professional life? Because this days means they have to take a pause form the job with possible consequencies.*
   - We apologize for the lack of clarity in the protocol at this point. Participants are encouraged to continue their daily occupation during the home challenge period and there are no restrictions on their activity during this time. Whe have clarified this; *Participants are otherwise encourage to continue their typical occupation during the challenge period with restriction.* (Added - Page 6 – Subsection - Treatment Arms - Home-Based Food Challenge (Intervention Arm))

Reviewer 3

1. *Will the patients have Epinephrine prescribed? – This should be clearly stated. If not all the patients will have the Epinephrine prescribed please state the reasons for prescribing or not.*
   a. Thank you for raising this important question. The decision to prescribe an adrenaline device to the patient is left at the discretion of the clinician reviewing the patient during their standard-of-care-appointment. This decision occurs before consideration of enrolment. This decision is made based on the characteristics of the previous food reaction, and the individual's context, such as co-morbidities, time to access emergency care, etc.
   b. Therefore, where patients report a history of food allergy where carriage of an adrenaline device is not typically recommended according to local guidelines (1) (e.g. isolated cutaneous symptoms), an adrenaline device will not be provided specifically for the study. This decision was informed

by the body of evidence indicating a negative impact on quality of life relating to adrenaline device prescription (2).

2. *I would suggest a phone call 48 hours after first dose and last dose ingestion to cover all possible AEs.*
    a. We appreciate the reviewer's comment and agree that protocolised phone calls would improve capture of AEs, however, regular calls during the home challenge period would also likely increase rates of adherence with the home protocol, and therefore overestimate the real-world feasibility of adherence to the home challenge protocol.
    b. In order to address both of these risks, phone calls after first and last dose ingestion are not currently protocolised, however participants are provided with a direct mobile number to a study team member and instructed to make contact in the event of any adverse events. This has now been included in the aforementioned trial manual.
3. *How would the patients avoiding multiple foods be handled? Who and how would chose the product for OFC in these cases?*
    a. Where participants have multiple food allergy labels that meet inclusion criteria for CHOMP, participant preference informs the selection of challenge food. This is stipulated in the protocol (page 12). This was instantiated to allow participant choice in proceeding with a particular challenge, in keeping with their personal values and preferences, rather than this being an investigator-led decision. After completion of the trial challenge, all participants are offered standard-of-care challenges for any other foods being avoided. We have clarified this within the article text;
        i. *Participants will be eligible to have one food allergy label assessed through this trial. Where an individual has multiple eligible food allergy labels, the selection of the food allergen to be investigated within this trial will be at the discretion of the participant. Further food allergy labels that remain at the end of the trial will be assessed as per standard of care.*

Summary of Changes

- *Participants are advised to abstain from exercise for 2 hours following each dose.* (Added - Page 6 – Subsection - Treatment Arms - Home-Based Food Challenge (Intervention Arm))
- *Participants are otherwise encourage to continue their typical occupation during the challenge period with restriction.* (Added - Page 6 – Subsection - Treatment Arms - Home-Based Food Challenge (Intervention Arm))
- *Avoid exercise in the 2 hours following each dose.* (Added - SUPPLEMENTARY FIGURE 2 – Home Challenge Protocol)

- Page 5, Line 41 - *'lale' corrected to 'label'*
- *Participants will be eligible to have one food allergy label assessed through this trial. Where an individual has multiple eligible food allergy labels, the selection of the food allergen to be investigated within this trial will be at the discretion of the participant. Further food allergy labels that remain at the end of the trial will be assessed as per standard of care.* (Added - Page 6 – Subsection - Treatment Arms - Home-Based Food Challenge (Intervention Arm))

References:

1. Australasian Society of Clinical Immunology and Allergy (ASCIA). (2026). ASCIA Action Plan: Allergic reactions [PDF]. Allergy & Anaphylaxis Australia. https://www.allergy.org.au/images/stories/anaphylaxis/2026/ASCIA_Action_Plan_Allergic_Reactions_2026.pdf
2. Pinczower GD, Bertalli NA, Bussmann N, Hamidon M, Allen KJ, DunnGalvin A, Hourihane JO, Gurrin LC, Tang ML. The effect of provision of an adrenaline autoinjector on quality of life in children with food allergy. J Allergy Clin Immunol. 2013 Jan;131(1):238-40.e1. doi: 10.1016/j.jaci.2012.09.038. Epub 2012 Nov 27. PMID: 23199601.
